# Crystal structure and Hirshfeld surface analysis of 2,4,6,11-tetra­kis­(4-fluoro­phen­yl)-9-oxa-1,5-di­aza­tri­cyclo­[5.3.1.0^3.8^]undeca­ne

**DOI:** 10.1107/S2056989018016122

**Published:** 2018-11-22

**Authors:** G. Vengatesh, M. Sundaravadivelu, Robert Swinton Darious

**Affiliations:** a Department of Chemistry, The Gandhigram Rural Institute – Deemed to be University, Gandhigram 624302, Tamilnadu, India; b School of Chemistry, Bharathidasan University, Tiruchirappalli 620 024, Tamilnadu, India

**Keywords:** crystal structure, di­aza­bicyclo bis­pidine, quinuclidine ring, Hirshfeld surface analysis, Fluoro­phen­yl, π-π stacking inter­actions

## Abstract

The title compound, C_32_H_26_F_4_N_2_O, crystallizes in the monoclinic space group *P*2_1_/*n* with four mol­ecules in the unit cell. The compound was prepared by the NaBH_4_ reduction of 4,8,9,10-tetra­kis­(4-fluoro­phen­yl)-1,3-di­aza­adamantan-6-one in chloro­form and ethanol as solvent. The piperidine rings exhibit chair and boat conformations, and all four fluoro­phenyl groups are oriented in the equatorial direction. The crystal structure features C—H⋯F hydrogen bonds, C—H⋯π, N—H⋯π and π–π inter­actions. Hirshfeld surface and two-dimensional fingerprint analysis show that van der Waals inter­actions constitute a major contribution to the inter­molecular inter­actions, with H⋯H contacts accounting for 37.9% of the surface.

## Abstract 2

The compound, prepared by the NaBH_4_ reduction of 4,8,9,10-tetra­kis­(4-fluoro­phen­yl)-1,3-di­aza­adamantan-6-one in chloro­form and ethanol as solvent, crystallizes in the monoclinic space group *P*2_1_/*n* with four mol­ecules in the unit cell.

## Chemical context   

Mol­ecules containing a bis­pidine nucleus are of great inter­est due to their presence in a wide variety of naturally occurring alkaloids and various biologically active mol­ecules (Jeyaraman & Avila, 1981 ▸). The biological activities of the mol­ecule depend crucially on the stereochemistry and conformation of the compound, and hence studies on the stereochemistry of the mol­ecules are inter­esting. The title compound contains four fluoro­phenyl groups and hence the investigation also looked for any weak inter­actions involving fluorine which are of current inter­est (Hathwar *et al.*, 2014 ▸). Moreover, Das *et al.* (2017 ▸) have recently discussed the role of halogens in stabilizing stacking patterns.
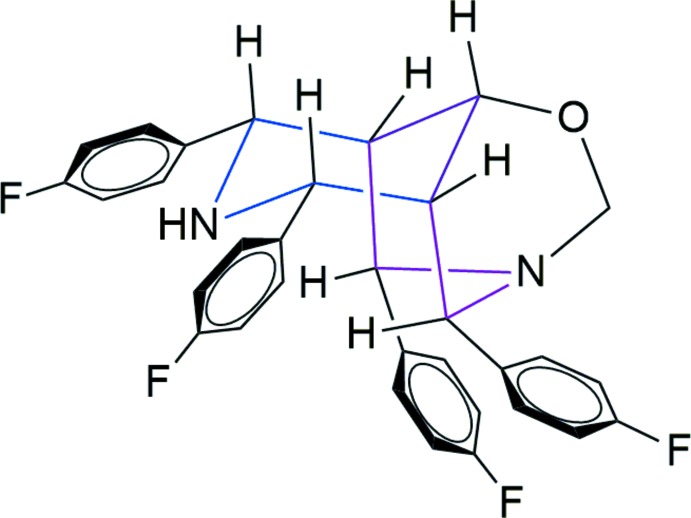


## Structural commentary   

**Figure 1:**
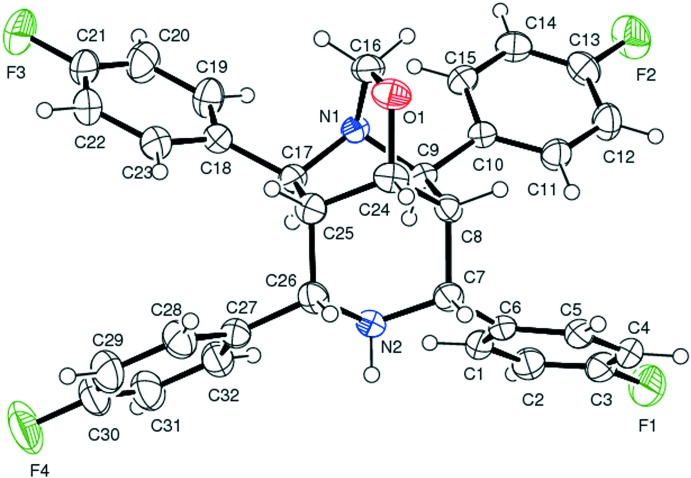
An *ORTEP* view of the title compound, showing the atom-numbering scheme. Displacement ellipsoids are drawn at 40% probability level.

An *ORTEP* view of the title compound is shown in Fig. 1 ▸. The N2/C7/C8/C24–C26 piperidine ring adopts a chair conformation with puckering parameters *Q* = 0.6178 (19) Å, θ = 176.85 (18)°, φ = 25 (3)° while the N1/C9/C8/C24/C25/C17 piperidine ring [puckering parameters *Q* = 0.8564 (18) Å, θ = 89.49 (12)°, φ = 178.52 (12)°] adopts a boat conformation. The oxygen-containing quinuclidine ring system (C8/C9/N1/C17/C25/C24/O1/C16) also adopts a boat conformation, with puckering parameters *Q* = 0.7817 (18) Å, θ = 91.23 (13)°, φ = 121.27 (13)° for the C8/C9/N1/C16/O1/C24 ring and *Q* = 0.7867 (18) Å, θ = 89.40 (13)°, φ = 297.43 (3)° for the C17/C25/C24/O1/C16/N1 ring. The fluoro­phenyl groups at C7 and C26 subtend a dihedral angle of 29.45 (1)° and are oriented equatorially with respect to the N2/C7/C8/C24–C26 piperidine ring with torsion angles C6—C7—C8—C24 = −179.72 (14)° and C24—C25—C26—C27 = 176.10 (14)°. The other two fluoro­phenyl groups at C9 and C17 subtend a dihedral angle of 21.85 (1)° and are oriented equatorially with respect to the N1/C9/C8/C24/C25/C17 piperidine ring, with torsion angles C10—C9—C8—C24 = 125.64 (15)° and C18—C17—C25—C24 = −128.24 (15)°.

## Supra­molecular features   

**Figure 2:**
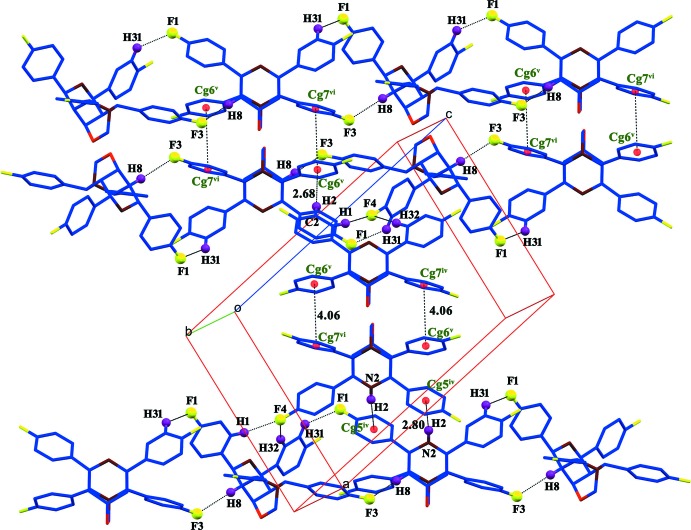
A view of the supra­molecular architecture of the title compound. Some of the atoms have been omitted for clarity.

In the crystal, several C—H⋯F hydrogen bonds occur. Screw-related mol­ecules are linked by C32—H32⋯F4^iii^ and C1—H1⋯F4^iii^ hydrogen bonds with F4 acting as a bifurcated acceptor (Table 1 ▸). The mol­ecules are further linked by C31—H31⋯F1^i^ and C8—H8⋯F3^ii^ hydrogen bonds (Fig. 2 ▸). An N—H⋯π inter­action is present along with intra- and inter­molecular C—H⋯π inter­actions (Table 1 ▸, Figs. 2 ▸ and 3 ▸). Weak π–π stacking inter­actions occur between the fluoro­phenyl groups [*Cg*6^v^⋯*Cg*7^vi^ = 4.0665 (12) Å; symmetry code: (vi) 1 − *x*, 1 − *y*, 1 − *z*; *Cg*6 and *Cg*7 are the centroids of the C10–C15 and C18–C23 rings respectively). Overall, these inter­actions generate a three-dimensional supra­molecular architecture.

## Hirshfeld surface analysis   

**Figure 4:**
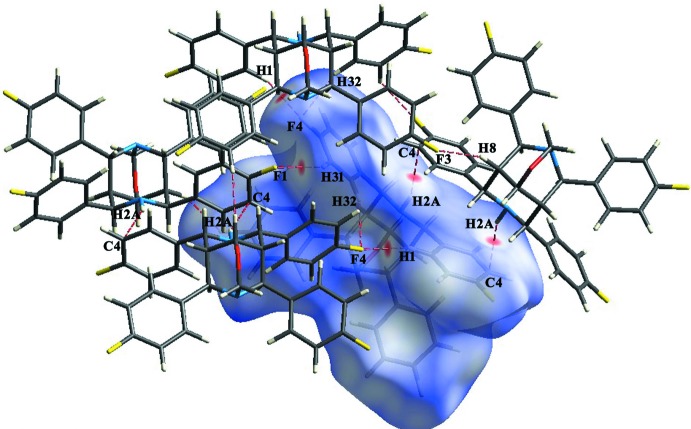
Hirshfeld surface of the title compound plotted over *d*_norm_, with neighbouring inter­actions shown as red dashed lines.

**Figure 5:**
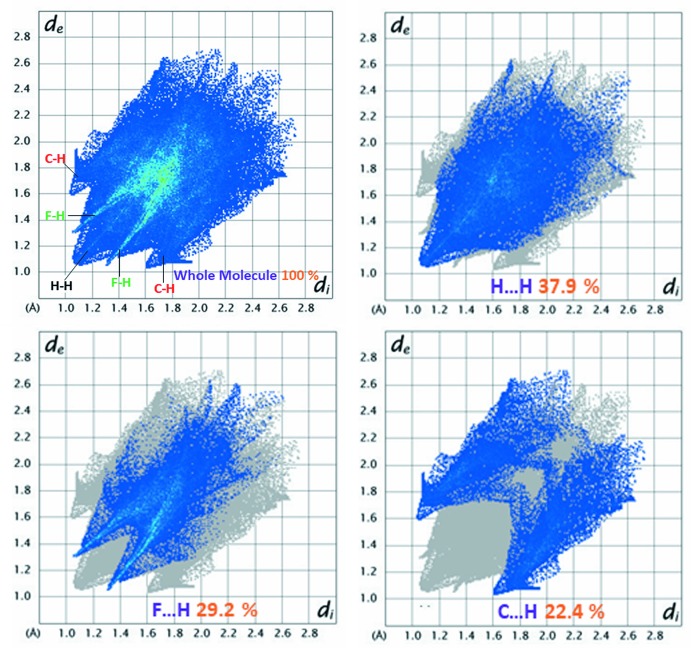
Two-dimensional fingerprint plots for the title compound.

Hirshfeld surface analysis and fingerprint plots, here generated with *Crystal Explorer* (Hirshfeld, 1977 ▸; Wolff *et al.*, 2012 ▸; Turner *et al.*, 2017 ▸), show the various inter­molecular inter­actions present in crystal structures (Wiedemann & Kohl, 2017 ▸; Tarahhomi *et al.*, 2013 ▸). Fig. 4 ▸ shows the Hirshfeld surface of the title compound mapped over *d*_norm_ where the intense red spots indicate regions of donor–acceptor inter­actions (Cárdenas-Valenzuela *et al.*, 2018 ▸; Atioğlu *et al.*, 2018 ▸) and represent the fluorine, carbon and hydrogen atoms involved. Fig. 5 ▸ shows the two-dimensional fingerprint plots, which qu­antify the contribution of each kind of inter­action to the surface formation (McKinnon *et al.*, 2007 ▸). The largest contribution to the surface of 37.9% is from H⋯H contacts, while C⋯H contacts contribute 22.4%; these represent van der Waals inter­actions present in the crystal. Inter­molecular hydrogen-bonding inter­actions (F⋯H/H⋯F contacts) contribute 29.2%.

## Database survey   

Di­aza­bicyclic compounds with different substituents on the aromatic rings have been reported in the literature: 2,4,6,8-tetra­kis­(4-ethyl­phen­yl)-3,7-di­aza­bicyclo-[3.3.1]-nonan-9-one [(I); Rajesh *et al.*, 2010 ▸], 2,4,6,8-tetra­kis­(4-bromo­phen­yl)-3,7di­aza­bicyclo-[3.3.1]-nonan-9-one [(II); Loh *et al.*, 2010 ▸], 2,4,6,8-tetra­kis­(2-meth­oxy­phen­yl)-3,7-di­aza­bicyclo­[3.3.1]non­an-9-one [(III); Fun *et al.*, 2009 ▸], 2,4,6,8-tetra­kis­(4-fluoro­phen­yl)-3,7-di­aza­bicyclo­[3.3.1]nonan-9-one [(IV); Natarajan *et al.*, 2008 ▸]. Compounds (I), (II) and (III) crystallize in space group *P*2_1_/*c*, while compound (IV) crystallizes in space group *C*2/*c*. The piperidine rings in all of these compounds adopt chair–boat conformations with an equatorial orientation of the aryl rings. In the crystal of (I), mol­ecules are linked via C—H⋯O hydrogen bonds, forming helical supra­molecular chains along the *b*-axis direction. In (II), the mol­ecules are connected through C—H⋯O and N—H⋯O hydrogen bonds, forming chains propagating along the *c*-axis direction, and C—H⋯π inter­actions also occur. The supra­molecular structure of compound (III) features C—H⋯N hydrogen bonds, which link the mol­ecules along the *b*-axis direction, and C—H⋯π inter­actions. In (IV), the mol­ecules are linked into a two-dimensional network by N—H⋯O, C—H⋯F and C—H⋯O hydrogen bonds and the crystal packing is further supported by N—H⋯π and C—H⋯π inter­actions.

Further background to the synthesis and stereochemistry of 3,7-di­aza­bicyclo­[3.3.1]nonan-9-ones and their derivatives can be seen in reports of the following structures: chloro­phenyl-1,3-di­aza­adamantan-6-one (Krishnakumar *et al.*, 2001 ▸), tetra­phenyl-1,3-di­aza­adamantan-6-one (Subha Nandhini *et al.*, 2002 ▸), fluoro­phenyl-1,3-di­aza­tri­cyclo­[3.3.1.1]decan-6-one (Natarajan *et al.*, 2009 ▸) and bis­pidine oxime (Parthiban *et al.*, 2010 ▸).

Weak C—H⋯F hydrogen bonds with similar bond lengths and bond angles to those in the title compound have been reported in the crystal structures of *N*-(3,5-di­fluoro­phen­yl)-9,10-di­hydro-9,10-ethano­anthracene-11,12-dicarboximide and *N*-(2,4,6-tri­fluoro­phen­yl)-9,10-di­hydro-9,10-ethano­anthra­cene-11,12-dicarboximide (Schwarzer & Weber, 2011 ▸), 2,3,5,6-tetra­fluoro­benzene-1,4-diol quinoxaline (Czapik & Gdaniec, 2010 ▸) and 2,3-di­fluoro-*N*-(4-pyrid­yl)benzamide (McMahon *et al.*, 2008 ▸). N—H⋯π inter­actions are present in the structures discussed by Fun *et al.* (2009 ▸) and Thirumurugan *et al.* (1999 ▸) while C—H⋯π inter­actions are present in the structures discussed by Selvanayagam *et al.* (2015 ▸), Muralikrishna *et al.* (2012 ▸) and Girisha *et al.* (2017 ▸).

## Synthesis and crystallization   

The title compound was synthesized in three steps starting from 4-fluoro­benzaldehyde, acetone and ammonium acetate. 4,8,9,10-Tetra­kis(4-fluoro­phen­yl)-1,3-di­aza­adamantan-6-one (1 mmol) dissolved in chloro­form and NaBH_4_ (1 mmol) dissolved in ethanol were mixed, transferred to a closed container and stirred at 278–283 K. The reaction was monitored by TLC, and after complete disappearance of the ketone the resulting mixture was filtered. The solvent was evaporated and washed with cold water to obtain the resulting product. The crude product was recrystallized from a chloro­form–ethanol (1:2 *v*:*v*) mixture by the solvent diffusion method.

## Refinement   

Crystal data, data collection and structure refinement details are summarized in Table 2 ▸. Carbon-bound hydrogen atoms were placed in calculated positions (C—H = 0.95–0.99 Å) and refined in the riding-model approximation with *U*_iso_(H) = 1.2–1.5*U*_eq_(C).

## Supplementary Material

Crystal structure: contains datablock(s) I. DOI: 10.1107/S2056989018016122/dx2010sup1.cif

Structure factors: contains datablock(s) I. DOI: 10.1107/S2056989018016122/dx2010Isup2.hkl

Supporting information file. DOI: 10.1107/S2056989018016122/dx2010Isup3.cml

CCDC reference: 1854381

Additional supporting information:  crystallographic information; 3D view; checkCIF report

## Figures and Tables

**Figure 3:**
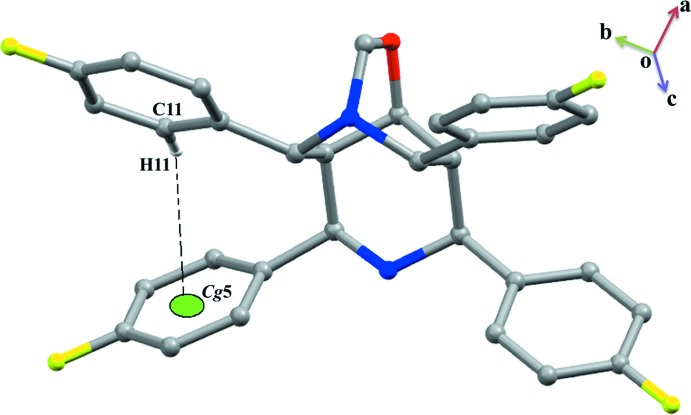
A view of the C11—H11⋯π inter­action (intra­molecular). Some of the atoms have been omitted for clarity.

**Table 1:** Hydrogen-bond geometry (Å, °) *Cg*5 and *Cg*6 are the centroids of the C1–C6 and C10–C15 rings, respectively.

*D*—H⋯*A*	*D*—H	H⋯*A*	*D*⋯*A*	*D*—H⋯*A*
C31—H31⋯F1^i^	0.93	2.51	3.231 (3)	135
C8—H8⋯F3^ii^	0.98	2.64	3.564 (2)	158
C32—H32⋯F4^iii^	0.93	2.66	3.567 (3)	162
C1—H1⋯F4^iii^	0.93	2.51	3.411 (2)	161
N2—H2*A*⋯*Cg*5^iv^	0.89	2.80 (2)	3.6594 (18)	161.7 (17)
C2—H2⋯*Cg*6^v^	0.93	2.68	3.552 (2)	156
C11—H11⋯*Cg*5	0.93	2.87	3.514 (2)	128

Symmetry codes: (i) 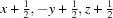; (ii) 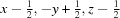; (iii) 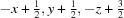; (iv) 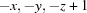; (v) 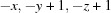.

**Table 2:** Experimental details

Crystal data	
Chemical formula	C_32_H_26_F_4_N_2_O
*M*_r_	530.55
Crystal system, space group	Monoclinic, *P*2_1_/*n*
Temperature (K)	296
*a*, *b*, *c* (Å)	13.5712 (8), 9.5161 (6), 20.1543 (13)
β (°)	99.357 (2)
*V* (Å^3^)	2568.2 (3)
*Z*	4
Radiation type	Mo *K*α
μ (mm^−1^)	0.10
Crystal size (mm)	0.15 × 0.10 × 0.10

Data collection	
Diffractometer	Bruker Kappa APEX3 CMOS
Absorption correction	Multi-scan (*SADABS*; Bruker, 2016 ▸)
*T*_min_, *T*_max_	0.711, 0.746
No. of measured, independent and observed [*I* > 2σ(*I*)] reflections	45339, 4510, 3456
*R*_int_	0.039
(sin θ/λ)_max_ (Å^−1^)	0.595

Refinement	
*R*[*F* ^2^ > 2σ(*F* ^2^)], *wR*(*F* ^2^), *S*	0.042, 0.117, 1.08
No. of reflections	4510
No. of parameters	356
H-atom treatment	H atoms treated by a mixture of independent and constrained refinement
Δρ_max_, Δρ_min_ (e Å^−3^)	0.17, −0.23

Computer programs: *APEX3*, *SAINT* and *XPREP* (Bruker, 2016 ▸), *SHELXT2014* (Sheldrick, 2015*a* ▸), *SHELXL2014* (Sheldrick, 2015*b* ▸), *ORTEP-3 for Windows* (Farrugia, 2012 ▸), *PLATON* (Spek, 2009 ▸), *Mercury* (Macrae *et al.*, 2008 ▸) and *publCIF* (Westrip, 2010 ▸).

## supplementary crystallographic information

### Crystal data

**Table:**

C_32_H_26_F_4_N_2_O	*F*(000) = 1104
*M**_r_* = 530.55	*D*_x_ = 1.372 Mg m^−^^3^
Monoclinic, *P*2_1_/*n*	Mo *K*α radiation, λ = 0.71073 Å
*a* = 13.5712 (8) Å	Cell parameters from 9987 reflections
*b* = 9.5161 (6) Å	θ = 2.9–27.2°
*c* = 20.1543 (13) Å	µ = 0.10 mm^−^^1^
β = 99.357 (2)°	*T* = 296 K
*V* = 2568.2 (3) Å^3^	Block, colourless
*Z* = 4	0.15 × 0.10 × 0.10 mm

### Data collection

**Table:**

Bruker Kappa APEX3 CMOS diffractometer	4510 independent reflections
Radiation source: fine-focus sealed tube	3456 reflections with *I* > 2σ(*I*)
Graphite monochromator	*R*_int_ = 0.039
ω and φ scan	θ_max_ = 25.0°, θ_min_ = 2.9°
Absorption correction: multi-scan (SADABS; Bruker, 2016)	*h* = −16→15
*T*_min_ = 0.711, *T*_max_ = 0.746	*k* = −11→11
45339 measured reflections	*l* = −23→23

### Refinement

**Table:**

Refinement on *F*^2^	0 restraints
Least-squares matrix: full	Hydrogen site location: mixed
*R*[*F*^2^ > 2σ(*F*^2^)] = 0.042	H atoms treated by a mixture of independent and constrained refinement
*wR*(*F*^2^) = 0.117	*w* = 1/[σ^2^(*F*_o_^2^) + (0.0488*P*)^2^ + 1.0821*P*] where *P* = (*F*_o_^2^ + 2*F*_c_^2^)/3
*S* = 1.08	(Δ/σ)_max_ < 0.001
4510 reflections	Δρ_max_ = 0.17 e Å^−^^3^
356 parameters	Δρ_min_ = −0.23 e Å^−^^3^

### Special details

**Table:**

Geometry. All esds (except the esd in the dihedral angle between two l.s. planes) are estimated using the full covariance matrix. The cell esds are taken into account individually in the estimation of esds in distances, angles and torsion angles; correlations between esds in cell parameters are only used when they are defined by crystal symmetry. An approximate (isotropic) treatment of cell esds is used for estimating esds involving l.s. planes.

### Fractional atomic coordinates and isotropic or equivalent isotropic displacement parameters (Å^2^)

**Table:**

	*x*	*y*	*z*	*U*_iso_*/*U*_eq_	
F4	0.36854 (15)	−0.15750 (19)	0.84036 (7)	0.1037 (6)	
F2	0.14924 (11)	0.80017 (13)	0.32630 (7)	0.0749 (4)	
F1	−0.19168 (9)	0.36154 (18)	0.39919 (8)	0.0810 (5)	
F3	0.71306 (11)	0.37803 (19)	0.77538 (7)	0.0862 (5)	
O1	0.43408 (9)	0.18807 (14)	0.44967 (6)	0.0460 (3)	
N1	0.36706 (10)	0.34033 (15)	0.52679 (7)	0.0333 (3)	
N2	0.19955 (12)	0.05682 (16)	0.54676 (7)	0.0367 (4)	
C30	0.3505 (2)	−0.1210 (3)	0.77425 (11)	0.0666 (7)	
C31	0.28511 (19)	−0.0145 (3)	0.75434 (11)	0.0629 (6)	
H31	0.2534	0.0331	0.7852	0.075*	
C32	0.26722 (16)	0.0210 (2)	0.68670 (10)	0.0490 (5)	
H32	0.2230	0.0935	0.6722	0.059*	
C27	0.31384 (14)	−0.04937 (19)	0.64048 (9)	0.0391 (4)	
C26	0.29591 (13)	−0.01240 (18)	0.56657 (9)	0.0375 (4)	
H26	0.2955	−0.0998	0.5408	0.045*	
C25	0.37815 (13)	0.08331 (18)	0.54726 (9)	0.0365 (4)	
H25	0.4432	0.0374	0.5596	0.044*	
C17	0.38155 (13)	0.23122 (18)	0.58041 (8)	0.0341 (4)	
H17	0.3236	0.2370	0.6036	0.041*	
C9	0.26532 (12)	0.32453 (17)	0.48748 (8)	0.0306 (4)	
H9	0.2189	0.3244	0.5200	0.037*	
C10	0.23762 (12)	0.44986 (18)	0.44154 (8)	0.0324 (4)	
C15	0.28174 (13)	0.58030 (19)	0.45697 (9)	0.0388 (4)	
H15	0.3311	0.5892	0.4945	0.047*	
C14	0.25352 (15)	0.6971 (2)	0.41748 (10)	0.0461 (5)	
H14	0.2852	0.7830	0.4273	0.055*	
C13	0.17844 (16)	0.6839 (2)	0.36394 (11)	0.0487 (5)	
C3	−0.10442 (14)	0.2911 (2)	0.41805 (11)	0.0526 (5)	
C4	−0.06943 (15)	0.2070 (2)	0.37201 (11)	0.0541 (6)	
H4	−0.1041	0.1985	0.3284	0.065*	
C5	0.01920 (15)	0.1348 (2)	0.39199 (9)	0.0449 (5)	
H5	0.0435	0.0761	0.3615	0.054*	
C6	0.07241 (13)	0.14846 (18)	0.45673 (8)	0.0354 (4)	
C1	0.03230 (13)	0.2329 (2)	0.50172 (9)	0.0415 (5)	
H1	0.0656	0.2412	0.5457	0.050*	
C2	−0.05616 (15)	0.3051 (2)	0.48247 (11)	0.0505 (5)	
H2	−0.0822	0.3620	0.5129	0.061*	
C7	0.17436 (13)	0.08262 (18)	0.47433 (9)	0.0352 (4)	
H7	0.1742	−0.0074	0.4507	0.042*	
C8	0.25592 (12)	0.17776 (17)	0.45258 (8)	0.0323 (4)	
H8	0.2418	0.1909	0.4037	0.039*	
C24	0.35693 (13)	0.10568 (18)	0.47129 (9)	0.0379 (4)	
H24	0.3543	0.0139	0.4490	0.045*	
C18	0.47282 (13)	0.2657 (2)	0.63240 (9)	0.0402 (4)	
C19	0.49034 (16)	0.4052 (2)	0.65186 (10)	0.0540 (5)	
H19	0.4467	0.4744	0.6322	0.065*	
C20	0.57105 (18)	0.4435 (3)	0.69963 (11)	0.0631 (6)	
H20	0.5824	0.5372	0.7116	0.076*	
C21	0.63320 (16)	0.3411 (3)	0.72859 (10)	0.0586 (6)	
C22	0.61911 (16)	0.2029 (3)	0.71244 (11)	0.0593 (6)	
H22	0.6625	0.1349	0.7335	0.071*	
C23	0.53842 (15)	0.1652 (2)	0.66377 (10)	0.0508 (5)	
H23	0.5284	0.0711	0.6521	0.061*	
C16	0.44100 (13)	0.3227 (2)	0.48368 (10)	0.0407 (4)	
H16A	0.5069	0.3319	0.5104	0.049*	
H16B	0.4332	0.3970	0.4503	0.049*	
C12	0.13179 (16)	0.5590 (2)	0.34729 (10)	0.0517 (5)	
H12	0.0805	0.5524	0.3107	0.062*	
C11	0.16255 (14)	0.4425 (2)	0.38611 (10)	0.0439 (5)	
H11	0.1319	0.3565	0.3747	0.053*	
C29	0.39832 (19)	−0.1925 (3)	0.73059 (13)	0.0674 (7)	
H29	0.4427	−0.2645	0.7457	0.081*	
C28	0.37981 (16)	−0.1566 (2)	0.66327 (11)	0.0538 (5)	
H28	0.4120	−0.2049	0.6329	0.065*	
H2A	0.1518 (16)	0.002 (2)	0.5589 (10)	0.057 (6)*	

### Atomic displacement parameters (Å^2^)

**Table:**

	*U*^11^	*U*^22^	*U*^33^	*U*^12^	*U*^13^	*U*^23^
F4	0.1449 (15)	0.1113 (13)	0.0448 (8)	−0.0144 (12)	−0.0148 (9)	0.0298 (8)
F2	0.0972 (10)	0.0440 (7)	0.0797 (9)	0.0120 (7)	0.0024 (8)	0.0233 (7)
F1	0.0451 (7)	0.1086 (12)	0.0845 (10)	0.0120 (8)	−0.0037 (7)	0.0280 (9)
F3	0.0702 (9)	0.1238 (13)	0.0541 (8)	−0.0221 (9)	−0.0216 (7)	−0.0030 (8)
O1	0.0436 (7)	0.0487 (8)	0.0510 (8)	0.0015 (6)	0.0231 (6)	−0.0002 (6)
N1	0.0292 (7)	0.0343 (8)	0.0361 (8)	−0.0024 (6)	0.0042 (6)	0.0022 (6)
N2	0.0369 (8)	0.0373 (8)	0.0358 (8)	−0.0050 (7)	0.0058 (7)	0.0065 (7)
C30	0.0842 (17)	0.0705 (16)	0.0392 (12)	−0.0193 (14)	−0.0072 (12)	0.0161 (12)
C31	0.0797 (16)	0.0687 (15)	0.0387 (12)	−0.0088 (13)	0.0051 (11)	0.0017 (11)
C32	0.0574 (12)	0.0465 (11)	0.0418 (11)	−0.0018 (10)	0.0040 (9)	0.0031 (9)
C27	0.0430 (10)	0.0343 (10)	0.0384 (10)	−0.0062 (8)	0.0014 (8)	0.0045 (8)
C26	0.0456 (10)	0.0289 (9)	0.0374 (10)	0.0002 (8)	0.0048 (8)	0.0006 (8)
C25	0.0355 (9)	0.0344 (9)	0.0403 (10)	0.0045 (8)	0.0082 (8)	0.0014 (8)
C17	0.0312 (9)	0.0348 (9)	0.0361 (9)	−0.0009 (7)	0.0050 (7)	0.0015 (8)
C9	0.0288 (9)	0.0298 (9)	0.0332 (9)	−0.0028 (7)	0.0054 (7)	−0.0014 (7)
C10	0.0309 (9)	0.0318 (9)	0.0352 (9)	−0.0008 (7)	0.0071 (7)	−0.0001 (7)
C15	0.0362 (10)	0.0370 (10)	0.0425 (10)	−0.0027 (8)	0.0048 (8)	−0.0042 (8)
C14	0.0531 (12)	0.0292 (9)	0.0575 (12)	−0.0037 (9)	0.0137 (10)	−0.0021 (9)
C13	0.0606 (13)	0.0354 (10)	0.0507 (12)	0.0085 (9)	0.0111 (10)	0.0114 (9)
C3	0.0327 (10)	0.0652 (14)	0.0579 (13)	−0.0047 (10)	0.0008 (9)	0.0164 (11)
C4	0.0461 (12)	0.0692 (14)	0.0419 (11)	−0.0212 (11)	−0.0077 (9)	0.0118 (11)
C5	0.0489 (11)	0.0487 (11)	0.0358 (10)	−0.0169 (9)	0.0032 (8)	−0.0011 (9)
C6	0.0359 (9)	0.0358 (9)	0.0335 (9)	−0.0123 (8)	0.0028 (7)	0.0024 (8)
C1	0.0361 (10)	0.0538 (12)	0.0340 (10)	−0.0063 (9)	0.0038 (8)	0.0005 (9)
C2	0.0390 (11)	0.0628 (13)	0.0504 (12)	0.0003 (10)	0.0096 (9)	0.0035 (10)
C7	0.0419 (10)	0.0290 (9)	0.0344 (9)	−0.0055 (8)	0.0053 (8)	−0.0032 (7)
C8	0.0380 (10)	0.0320 (9)	0.0273 (8)	−0.0023 (7)	0.0066 (7)	−0.0002 (7)
C24	0.0416 (10)	0.0319 (9)	0.0427 (10)	−0.0007 (8)	0.0148 (8)	−0.0039 (8)
C18	0.0373 (10)	0.0484 (11)	0.0347 (10)	−0.0038 (8)	0.0055 (8)	0.0036 (9)
C19	0.0564 (13)	0.0536 (13)	0.0476 (12)	0.0015 (10)	−0.0054 (10)	−0.0073 (10)
C20	0.0687 (15)	0.0675 (15)	0.0482 (13)	−0.0104 (12)	−0.0056 (11)	−0.0130 (11)
C21	0.0504 (13)	0.0873 (18)	0.0347 (11)	−0.0140 (12)	−0.0034 (9)	0.0008 (11)
C22	0.0484 (12)	0.0773 (17)	0.0479 (12)	−0.0026 (11)	−0.0051 (10)	0.0197 (12)
C23	0.0480 (12)	0.0522 (12)	0.0492 (12)	−0.0047 (10)	−0.0006 (9)	0.0116 (10)
C16	0.0345 (10)	0.0416 (10)	0.0471 (11)	−0.0026 (8)	0.0100 (8)	0.0053 (9)
C12	0.0570 (13)	0.0461 (12)	0.0469 (12)	0.0017 (10)	−0.0068 (10)	0.0057 (9)
C11	0.0474 (11)	0.0350 (10)	0.0464 (11)	−0.0068 (8)	−0.0016 (9)	0.0021 (8)
C29	0.0718 (16)	0.0562 (14)	0.0665 (16)	0.0019 (12)	−0.0117 (13)	0.0235 (12)
C28	0.0581 (13)	0.0465 (12)	0.0543 (13)	0.0040 (10)	0.0016 (10)	0.0114 (10)

### Geometric parameters (Å, º)

**Table:**

F4—C30	1.360 (2)	C13—C12	1.362 (3)
F2—C13	1.364 (2)	C3—C2	1.362 (3)
F1—C3	1.360 (2)	C3—C4	1.367 (3)
F3—C21	1.362 (2)	C4—C5	1.387 (3)
O1—C24	1.431 (2)	C4—H4	0.9300
O1—C16	1.449 (2)	C5—C6	1.391 (3)
N1—C16	1.440 (2)	C5—H5	0.9300
N1—C9	1.484 (2)	C6—C1	1.387 (3)
N1—C17	1.488 (2)	C6—C7	1.508 (3)
N2—C26	1.461 (2)	C1—C2	1.383 (3)
N2—C7	1.465 (2)	C1—H1	0.9300
N2—H2A	0.89 (2)	C2—H2	0.9300
C30—C29	1.358 (4)	C7—C8	1.548 (2)
C30—C31	1.364 (4)	C7—H7	0.9800
C31—C32	1.387 (3)	C8—C24	1.524 (2)
C31—H31	0.9300	C8—H8	0.9800
C32—C27	1.381 (3)	C24—H24	0.9800
C32—H32	0.9300	C18—C23	1.387 (3)
C27—C28	1.385 (3)	C18—C19	1.394 (3)
C27—C26	1.511 (2)	C19—C20	1.384 (3)
C26—C25	1.539 (2)	C19—H19	0.9300
C26—H26	0.9800	C20—C21	1.357 (3)
C25—C24	1.526 (2)	C20—H20	0.9300
C25—C17	1.555 (2)	C21—C22	1.361 (3)
C25—H25	0.9800	C22—C23	1.393 (3)
C17—C18	1.522 (2)	C22—H22	0.9300
C17—H17	0.9800	C23—H23	0.9300
C9—C10	1.519 (2)	C16—H16A	0.9700
C9—C8	1.560 (2)	C16—H16B	0.9700
C9—H9	0.9800	C12—C11	1.382 (3)
C10—C11	1.386 (3)	C12—H12	0.9300
C10—C15	1.391 (2)	C11—H11	0.9300
C15—C14	1.384 (3)	C29—C28	1.382 (3)
C15—H15	0.9300	C29—H29	0.9300
C14—C13	1.364 (3)	C28—H28	0.9300
C14—H14	0.9300		
			
C24—O1—C16	109.55 (12)	C1—C6—C5	117.92 (17)
C16—N1—C9	110.20 (14)	C1—C6—C7	121.96 (15)
C16—N1—C17	109.51 (14)	C5—C6—C7	119.91 (17)
C9—N1—C17	108.58 (12)	C2—C1—C6	121.29 (18)
C26—N2—C7	113.67 (14)	C2—C1—H1	119.4
C26—N2—H2A	108.7 (14)	C6—C1—H1	119.4
C7—N2—H2A	107.9 (14)	C3—C2—C1	118.7 (2)
C29—C30—F4	118.5 (2)	C3—C2—H2	120.7
C29—C30—C31	122.6 (2)	C1—C2—H2	120.7
F4—C30—C31	118.9 (3)	N2—C7—C6	111.15 (14)
C30—C31—C32	118.1 (2)	N2—C7—C8	108.58 (14)
C30—C31—H31	120.9	C6—C7—C8	111.19 (14)
C32—C31—H31	120.9	N2—C7—H7	108.6
C27—C32—C31	121.3 (2)	C6—C7—H7	108.6
C27—C32—H32	119.4	C8—C7—H7	108.6
C31—C32—H32	119.4	C24—C8—C7	108.82 (14)
C32—C27—C28	118.39 (18)	C24—C8—C9	106.68 (13)
C32—C27—C26	122.27 (17)	C7—C8—C9	113.95 (13)
C28—C27—C26	119.34 (17)	C24—C8—H8	109.1
N2—C26—C27	111.59 (15)	C7—C8—H8	109.1
N2—C26—C25	108.53 (14)	C9—C8—H8	109.1
C27—C26—C25	112.33 (15)	O1—C24—C8	110.56 (14)
N2—C26—H26	108.1	O1—C24—C25	110.71 (14)
C27—C26—H26	108.1	C8—C24—C25	109.05 (14)
C25—C26—H26	108.1	O1—C24—H24	108.8
C24—C25—C26	108.06 (14)	C8—C24—H24	108.8
C24—C25—C17	106.99 (14)	C25—C24—H24	108.8
C26—C25—C17	113.51 (14)	C23—C18—C19	117.44 (18)
C24—C25—H25	109.4	C23—C18—C17	123.74 (18)
C26—C25—H25	109.4	C19—C18—C17	118.77 (17)
C17—C25—H25	109.4	C20—C19—C18	121.7 (2)
N1—C17—C18	110.25 (14)	C20—C19—H19	119.1
N1—C17—C25	109.16 (13)	C18—C19—H19	119.1
C18—C17—C25	117.03 (15)	C21—C20—C19	118.5 (2)
N1—C17—H17	106.6	C21—C20—H20	120.8
C18—C17—H17	106.6	C19—C20—H20	120.8
C25—C17—H17	106.6	C20—C21—C22	122.5 (2)
N1—C9—C10	111.25 (13)	C20—C21—F3	118.8 (2)
N1—C9—C8	109.41 (13)	C22—C21—F3	118.7 (2)
C10—C9—C8	115.73 (13)	C21—C22—C23	118.8 (2)
N1—C9—H9	106.6	C21—C22—H22	120.6
C10—C9—H9	106.6	C23—C22—H22	120.6
C8—C9—H9	106.6	C18—C23—C22	121.1 (2)
C11—C10—C15	117.29 (16)	C18—C23—H23	119.5
C11—C10—C9	121.89 (15)	C22—C23—H23	119.5
C15—C10—C9	120.62 (15)	N1—C16—O1	112.99 (14)
C14—C15—C10	121.28 (17)	N1—C16—H16A	109.0
C14—C15—H15	119.4	O1—C16—H16A	109.0
C10—C15—H15	119.4	N1—C16—H16B	109.0
C13—C14—C15	118.81 (18)	O1—C16—H16B	109.0
C13—C14—H14	120.6	H16A—C16—H16B	107.8
C15—C14—H14	120.6	C13—C12—C11	118.35 (19)
C12—C13—F2	119.30 (19)	C13—C12—H12	120.8
C12—C13—C14	122.19 (18)	C11—C12—H12	120.8
F2—C13—C14	118.50 (18)	C12—C11—C10	122.04 (18)
F1—C3—C2	118.7 (2)	C12—C11—H11	119.0
F1—C3—C4	118.78 (19)	C10—C11—H11	119.0
C2—C3—C4	122.5 (2)	C30—C29—C28	118.8 (2)
C3—C4—C5	118.24 (19)	C30—C29—H29	120.6
C3—C4—H4	120.9	C28—C29—H29	120.6
C5—C4—H4	120.9	C29—C28—C27	120.8 (2)
C4—C5—C6	121.31 (19)	C29—C28—H28	119.6
C4—C5—H5	119.3	C27—C28—H28	119.6
C6—C5—H5	119.3		
			
C29—C30—C31—C32	0.1 (4)	C5—C6—C7—N2	−156.40 (16)
F4—C30—C31—C32	−179.7 (2)	C1—C6—C7—C8	−92.14 (19)
C30—C31—C32—C27	0.1 (3)	C5—C6—C7—C8	82.51 (19)
C31—C32—C27—C28	−0.3 (3)	N2—C7—C8—C24	57.70 (17)
C31—C32—C27—C26	−179.76 (19)	C6—C7—C8—C24	−179.72 (14)
C7—N2—C26—C27	−174.43 (14)	N2—C7—C8—C9	−61.20 (18)
C7—N2—C26—C25	61.26 (18)	C6—C7—C8—C9	61.39 (18)
C32—C27—C26—N2	−23.9 (2)	N1—C9—C8—C24	−0.98 (17)
C28—C27—C26—N2	156.61 (17)	C10—C9—C8—C24	125.64 (15)
C32—C27—C26—C25	98.2 (2)	N1—C9—C8—C7	119.13 (15)
C28—C27—C26—C25	−81.2 (2)	C10—C9—C8—C7	−114.26 (16)
N2—C26—C25—C24	−60.04 (18)	C16—O1—C24—C8	62.17 (18)
C27—C26—C25—C24	176.10 (14)	C16—O1—C24—C25	−58.79 (18)
N2—C26—C25—C17	58.46 (19)	C7—C8—C24—O1	177.82 (13)
C27—C26—C25—C17	−65.41 (19)	C9—C8—C24—O1	−58.83 (17)
C16—N1—C17—C18	73.99 (17)	C7—C8—C24—C25	−60.24 (18)
C9—N1—C17—C18	−165.64 (14)	C9—C8—C24—C25	63.11 (17)
C16—N1—C17—C25	−55.89 (17)	C26—C25—C24—O1	−176.91 (13)
C9—N1—C17—C25	64.48 (16)	C17—C25—C24—O1	60.51 (17)
C24—C25—C17—N1	−2.18 (18)	C26—C25—C24—C8	61.24 (18)
C26—C25—C17—N1	−121.29 (15)	C17—C25—C24—C8	−61.34 (17)
C24—C25—C17—C18	−128.24 (15)	N1—C17—C18—C23	−142.50 (18)
C26—C25—C17—C18	112.65 (17)	C25—C17—C18—C23	−17.0 (3)
C16—N1—C9—C10	−71.76 (17)	N1—C17—C18—C19	40.0 (2)
C17—N1—C9—C10	168.30 (13)	C25—C17—C18—C19	165.47 (17)
C16—N1—C9—C8	57.36 (17)	C23—C18—C19—C20	1.3 (3)
C17—N1—C9—C8	−62.58 (16)	C17—C18—C19—C20	178.99 (19)
N1—C9—C10—C11	159.75 (16)	C18—C19—C20—C21	−1.1 (4)
C8—C9—C10—C11	34.1 (2)	C19—C20—C21—C22	0.0 (4)
N1—C9—C10—C15	−25.5 (2)	C19—C20—C21—F3	179.7 (2)
C8—C9—C10—C15	−151.19 (16)	C20—C21—C22—C23	0.7 (4)
C11—C10—C15—C14	−1.6 (3)	F3—C21—C22—C23	−178.93 (18)
C9—C10—C15—C14	−176.61 (16)	C19—C18—C23—C22	−0.5 (3)
C10—C15—C14—C13	2.4 (3)	C17—C18—C23—C22	−178.11 (18)
C15—C14—C13—C12	−1.4 (3)	C21—C22—C23—C18	−0.4 (3)
C15—C14—C13—F2	178.30 (17)	C9—N1—C16—O1	−57.81 (19)
F1—C3—C4—C5	−179.50 (17)	C17—N1—C16—O1	61.56 (18)
C2—C3—C4—C5	−0.7 (3)	C24—O1—C16—N1	−2.7 (2)
C3—C4—C5—C6	−0.9 (3)	F2—C13—C12—C11	−179.99 (19)
C4—C5—C6—C1	2.2 (3)	C14—C13—C12—C11	−0.3 (3)
C4—C5—C6—C7	−172.63 (17)	C13—C12—C11—C10	1.1 (3)
C5—C6—C1—C2	−2.0 (3)	C15—C10—C11—C12	−0.1 (3)
C7—C6—C1—C2	172.73 (17)	C9—C10—C11—C12	174.80 (18)
F1—C3—C2—C1	179.71 (18)	F4—C30—C29—C28	179.6 (2)
C4—C3—C2—C1	0.9 (3)	C31—C30—C29—C28	−0.3 (4)
C6—C1—C2—C3	0.5 (3)	C30—C29—C28—C27	0.1 (3)
C26—N2—C7—C6	177.57 (14)	C32—C27—C28—C29	0.2 (3)
C26—N2—C7—C8	−59.82 (18)	C26—C27—C28—C29	179.65 (19)
C1—C6—C7—N2	28.9 (2)		

### Hydrogen-bond geometry (Å, º)

Cg5 and Cg6 are the centroids of the C1–C6 and C10–C15 rings, respectively.

**Table:**

*D*—H···*A*	*D*—H	H···*A*	*D*···*A*	*D*—H···*A*
C31—H31···F1^i^	0.93	2.51	3.231 (3)	135
C8—H8···F3^ii^	0.98	2.64	3.564 (2)	158
C32—H32···F4^iii^	0.93	2.66	3.567 (3)	162
C1—H1···F4^iii^	0.93	2.51	3.411 (2)	161
N2—H2*A*···*Cg*5^iv^	0.89	2.80 (2)	3.6594 (18)	161.7 (17)
C2—H2···*Cg*6^v^	0.93	2.68	3.552 (2)	156
C11—H11···*Cg*5	0.93	2.87	3.514 (2)	128

Symmetry codes: (i) *x*+1/2, −*y*+1/2, *z*+1/2; (ii) *x*−1/2, −*y*+1/2, *z*−1/2; (iii) −*x*+1/2, *y*+1/2, −*z*+3/2; (iv) −*x*, −*y*, −*z*+1; (v) −*x*, −*y*+1, −*z*+1.
